# The Laser Vegetation Detecting Sensor: A Full Waveform, Large-Footprint, Airborne Laser Altimeter for Monitoring Forest Resources

**DOI:** 10.3390/s19071699

**Published:** 2019-04-10

**Authors:** Yang Hu, Fayun Wu, Zhongqiu Sun, Andrew Lister, Xianlian Gao, Weitao Li, Daoli Peng

**Affiliations:** 1 College of Forestry, Beijing Forestry University, Beijing 100083, China; huyang@bjfu.edu.cn; 2 Academy of Forest Inventory and Planning, National Forestry and Grassland Administration, Beijing 100714, China; wufayun@sina.com (F.W.); qiuqiu8708@163.com (Z.S.); gaoxianlian001@163.com (X.G.); 3 USDA Forest Service, Northern Research Station, Newtown Square, PA 19073, USA; andylister100@gmail.com; 4 Geography Information and Tourism College, Chuzhou University, Chuzhou 239000, China; liweitao_801225@126.com

**Keywords:** forest inventory, airborne sensors, large-footprint LiDAR, LiDAR design, LiDAR application

## Abstract

The use of satellite-borne large-footprint LiDAR (light detection and ranging) systems allows for the acquisition of forest monitoring data. This paper mainly describes the design, use, operating principles, installation and data properties of the new Laser Vegetation Detecting Sensor (LVDS), a LiDAR system designed and developed at the Academy of Forest Inventory and Planning (AFIP) and the Beijing Institute of Telemetry (BIT). Data from LVDS were used to calculate the mean height of forest trees on sample plots using data collected in the Hunan province of China. The results show that the full waveform data obtained by LVDS has the ability to accurately characterize forest height. The mean absolute percentage error of mean forest height per plot in flat areas was 6.8%, with a mean absolute deviation of 0.78 m. The airborne LVDS system provides prototype data sets and a platform for instrument proof-of-concept studies for China’s Terrestrial Ecosystem Carbon Monitoring (TECM) mission, which is an Earth remote sensing satellite due for launch in 2020. The information produced by LVDS allows for forest structure studies with high accuracy and coverage of large areas.

## 1. Introduction

Forests play an important role in the ecosystem. With the development of China’s economy, forest resource monitoring has become more and more important as pressures on forest resources have increased. Mean canopy height is an important parameter that reflects the vertical structure of forests [1,2], and is of great importance for the quantitative estimation of forest habitat extent [3], biomass quantity, and the area affected by landscape changes [4,5,6]. In traditional field-based forest surveys, ground measurements on inventory plots have been used to estimate mean forest height. Not only is this method time- and effort-consuming, but also tree height collection is often unreliable, especially in areas where forests are dense or otherwise difficult to traverse. Therefore, traditional forest survey instruments cannot meet the need for low-cost, reliable forest canopy height information.

Optical remote sensing images from passive sensors have been used extensively to estimate the horizontal distribution of forest. For example, spaceborne sensor systems like the Landsat series [7,8,9], MODIS [10,11,12,13], GF series [14,15,16], ZY-3 [17,18], and Sentinel series [16,19,20] provide spectral information that is used as the foundation for image classification globally. However, due to the complex spatial structure of forests and limitations of optical data, forest cover maps from these sources often suffer from inaccuracy [21], particularly for the classification of vertical forest structure [22]. Microwave remote sensing, a technology that uses an active sensor system, has often been used for tree height estimation, but its signal tends to saturate in forests with dense canopies [23]. Compared to other technologies, light detection and ranging (LiDAR) have been shown to be very useful for detecting vertical structural attributes like canopy height and tree crown density in forested areas [24]. The advanced technology, coupled with the GNSS (Global Navigation Satellite System) and IMU (inertial measurement unit), can improve the horizontal and vertical positional accuracy of airborne remote sensing data to the decimeter level [25].

The first Scanning LiDAR Imager of Canopies by Echo Recovery (SLICER), which had a 15 m footprint, was designed by NASA in the early 1990s. A series of studies about the equipment and data products followed [26,27,28,29]. A later advance in laser remote sensing was the launch of the Laser Vegetation Imaging Sensor (LVIS) [30], designed as the airborne simulator for the Vegetation Canopy LiDAR (VCL) mission. Performance tests and analyses of the properties of these data were conducted, leading to a growth in interest in the use of laser imaging technology [31]. In January 2003, the Geoscience Laser Altimeter System (GLAS), carried on the ICESat instrument, was launched. This sensor provided full waveform LiDAR data for a set of well-distributed, 70-m diameter footprints for much of the globe [32,33]. GLAS data were used in various ways to calibrate models of vegetation structure parameters [34,35,36,37,38,39,40,41]. The Global Ecosystem Dynamics Investigation (GEDI), carried by the SpaceX Commercial Resupply Mission 16, launched successfully on December 5th, 2018 from Cape Canaveral in Florida. GEDI will map ecosystem structures including carbon and nutrient cycling, habitat quality and biodiversity, forest health and productivity, hydrologic cycling, and effects of natural and human caused disturbances. At the same time, some researchers have proved the GEDI’s ability to estimate ground elevation and forest parameters (maximum forest height and forest aboveground biomass) through simulated GEDI data [42,43,44,45].

The successful application of spaceborne LiDAR in forest monitoring provides an impetus for researchers in China to improve the country’s forest resource monitoring systems. Motivated by this interest, the Terrestrial Ecosystem Carbon Monitoring Satellite Project, which consists of a large-footprint LiDAR system, was formally approved. The mission will launch at the end of 2020, with the goal of providing continuous forest resource monitoring of China. In order to test the sensor system prior to implementation of the spaceborne instrument, a ¼ scale, large-footprint airborne LiDAR system based on the spaceborne system was designed by the Academy of Forest Inventory and Planning, State Forestry Administration of China.

To the best of our knowledge, no study has introduced this new airborne large-footprint LiDAR system and tested the application of the system to estimate characteristics of different landscape features such as forest height. The aim of this study, therefore, was to deploy and test a newly designed large-footprint LiDAR system that was developed to test technology that will eventually be deployed in space. In forested areas, we examined the relationship between LiDAR height estimates and mean forest height based on ground plot data. Our goal was to better understand the capabilities of the sensor and to develop a validation procedure for this type of data.

## 2. The Laser Vegetation Detecting Sensor (LVDS)

### 2.1. System Overview

**Figure 1:**
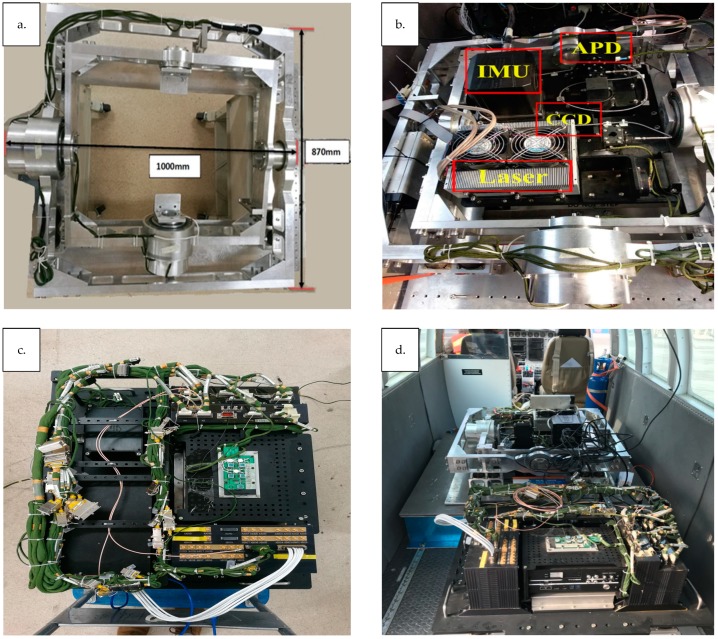
(**a**) Two-dimensional stabilized platform; (**b**) laser, CCD, IMU, and APD are installed in (**a**); (**c**) signal processing and control unit; (**a**,**c**) are mounted on the airplane adjacent to one another (**d**).

LVDS is a pulsed laser altimeter, which measures the distance between the instrument and the target surface. The entire time history of the outgoing and return laser pulses is digitized using a single detector, digital converter, and timing clock to clearly describe the vertical distribution of surfaces within each laser footprint. The main system is composed of a laser sensor unit, a signal processing and control unit, a two-dimensional stabilized platform, a GNSS/INS integrated navigation unit, and a charge coupled device (CCD) camera unit (Figure 1).

The signal processing and control unit controls the emissions from the single-wavelength solid state laser, as well as conducts proportional beam splitting of the transmitted beam through the optical beam splitting device. A low energy beam is coupled to the relay optical device through a fiber optic cable. The laser beam is then coupled to the detector by the coupling lens of the relay optical device. A high energy beam is then expanded and sent to the ground by the transmitting optical beam splitting device. The laser light signal reflected by the ground targets is then received by the telescope. This received signal is filtered and focused by the relay optical device, and the detector transforms the laser light signals into electrical signals.

The signal processing and control unit collects, processes and uploads these electrical signals, collects data on the working state of system, and sends these to the real-time processing and display software of the upper computer. The upper computer’s real-time processing and display software integrates and processes the received signals from the sensors and control unit, allowing for real-time data display and storage.

The GNSS/INS combined navigation unit and laser sensor unit are affixed to the frame of the unit and used to obtain the current position and attitude data of the laser sensor unit in real-time. The laser sensor unit and aerial camera are mounted on the two-dimensional stabilized platform. The two-dimensional stabilized platform holds the gyroscope sensor and laser sensor units. Based on the real-time attitude data of the gyroscope sensor, the two-dimensional stabilized platform attitude is adjusted dynamically, so as to keep the appropriate pointing angle of the laser and to absorb shock experienced by the laser sensor unit. The aerial camera points in the same direction as the laser sensor unit to complete the image acquisition of ground targets.

#### 2.1.1. Laser Sensor Unit

**Figure 2:**
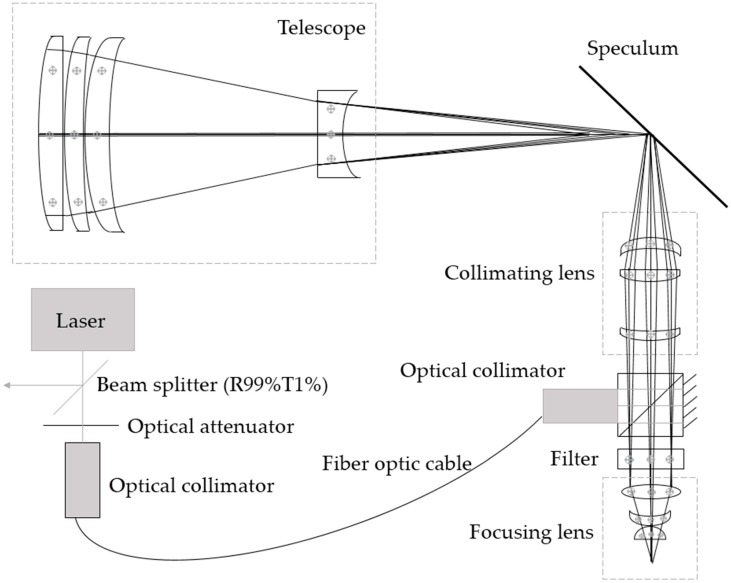
Laser sensor unit design.

The laser sensor unit (Figure 2) consists of a single-wavelength solid-state laser, a transmitting optical beam splitting device, a telescope, a relay optical device, and a detector. By applying narrow pulse laser emission technology, the single-pulse energy is reduced and the echo signal-to-noise ratio will be high. At the same time, the overall application safety and reliability of the system can be improved, and the volume of the system (1000 × 870 × 550 mm) can be effectively reduced. The output wavelength of the laser is 1064 nm, the single-pulse energy is 2 mJ, the pulse repetition frequency is 40 Hz, and the pulse width is 1.5 ns. The beam splitting ratio of the transmitting optical beam splitting device is 1:100. The divergence angle of the emitting laser beam after beam expansion is 5 mrad, and the footprint size is 15 m at altitudes up to 3 km above ground level.

The receiving telescope adopts the transmitted configuration, with the advantage of no center shielding in the field of view, high optical efficiency, compact structure, and reducing the difficulty of machining and setting. The telescope consists of four BK7 lenses, with an effective diameter of 100 mm, a total length of 298.8 mm, a receiving field of view angle of 6 mrad, an effective focal length of 148.8 mm, and an image surface diameter of 3 mm.

The relay optical unit is mainly composed of a speculum, a collimating lens, a focusing lens and a detector triggering light path. The system length is shortened by placing a speculum in front of the focal length of the telescope. The optical spot diameter of the telescope mirror surface is about 3 mm, and the diameter of the photosensitive surface of the detector is about 0.8 mm. Collimating and focusing the echo signal received by the telescope can reduce energy loss and improve the detection efficiency of the system. The detector in the relay optical unit triggers the light path, processes the energy introduced by the fiber optic, and triggers the detector. The field of view of the collimating lens was designed to be ±20 mrad, and the diameter of the image surface (3 mm) is consistent with that of the telescope mirror surface. The collimating lens is composed of three BK7 lenses, which has a total length of about 100 mm and a posterior intercept of 62 mm. The imaging quality is close to the diffraction limit. The function of the focus lens is to focus all echo signals into the photosensitive surface of the detector. The focus lens is made up of three BK7 lenses, with a total length of about 25 mm and a diameter of 0.3 mm for the light spot on the image surface. The trigger optical channel can adjust the trigger energy, simplify the structure design and shield stray light interference by placing a customized prism at the rear end of the collimating lens.

The detector is used to convert the laser echoes received by the optical receiving system into electrical signals for the measurement of signal amplitude, waveform and flight time. It uses a silicon avalanche diode photoelectric receiving module as the detection unit. A semiconductor cooling unit is integrated into the sealed package to improve the heat dissipation performance and stability of the detector. The diameter of the photosensitive surface of the detector is 0.8 mm, and its responsiveness to the wavelength of 1064 nm is 200 KV/W.

#### 2.1.2. Signal Processing and Control Unit

**Figure 3:**
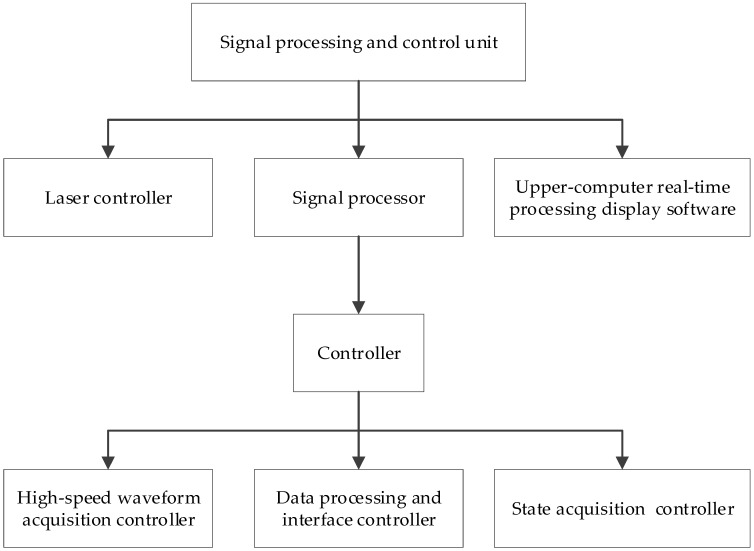
Signal processing and control unit structure.

The signal processing and control unit (Figure 3) is composed of a high-speed waveform acquisition collector, data processing and interface controller, state acquisition controller, controller, upper-computer real-time processing display software, and a laser controller. All units are integrated into a single structural platform, which effectively reduces the number and complexity of devices and improves their reliability, convenience, and usability.

The laser controller controls the laser’s emission time, frequency, and energy. The controller is responsible for the signal processing and control unit, command sending and receiving, data storage, and output of synchronous signals. The data storage capacity can reach 1 TB. The high-speed waveform collector completes high-speed sampling of the electrical signal output by the detector, triggered by the system synchronization signal output by the controller. The sampling rate is 1 GHz, and the effective sampling bits can reach 10 bits. The data processing and interface control complete the processing and packaging of sampled data, and upload measurement data in real-time through an ethernet interface and controller. The state acquisition and controller are used to monitor the system state in real time and complete the setting of working parameters and uploading of working state detection. The upper-computer real-time processing display software is installed in the controller system for system parameter configuration, system switching machine control, real-time display of state monitoring parameters, and real-time display of sampled waveforms.

#### 2.1.3. Two-Dimensional Stability Platform

The two-dimensional stability platform is designed with a two-dimensional orthogonal rotating shaft structure. The two-way rotating shaft is driven by an independent servo motor. The system integrates the two-axis gyro inertial sensor (INS) and attitude automatic controller, which can measure the attitude change of the laser radar installation platform in real time and calculate the motor rotation angle to be adjusted in real time. The two-dimensional stability platform can respond quickly to the attitude change of the system platform to ensure the stability of the system during the measurement process. At the same time, the shock absorber is connected at the bottom of the high stability platform to filter high-frequency vibration due to aircraft flight motion and reduce its impact on measurements. The stability range (azimuth and pitching axis) is greater than or equal to ±8 deg, the maximum angular velocity (azimuth and pitching axis) is greater than or equal to 50 deg/s, the maximum angular acceleration (azimuth and pitching axis) is greater than or equal to 200 deg/s^2^, the dynamic error (azimuth and pitching axis) is less than 0.08 deg (3 deg, 3 Hz), the vertical deviation is less than 0.01 deg in automatic leveling mode and the effective load is 50 kg.

#### 2.1.4. GNSS/INS Integrated Navigation Unit

The INS module provides platform roll, pitch, and bearing information at a 200 Hz sampling rate with 0.005 deg resolution. The GNSS module provides real-time geographic position of the instrument which is fed into a pilot assistance system that allows precision flying (to within 0.008 deg) along predetermined flight tracks. Data from the on-board GNSS are processed post-flight with data from ground-based, GNSS receivers to provide an airplane trajectory with vertical accuracy of ~30 cm.

#### 2.1.5. CCD Camera Unit

A Phase One iXU-R180 was selected as the aerial camera. Its main performance parameters are shown in Table 1. Further details on the camera specifications can be found at https://industrial.phaseone.com/.

## 3. Calibration of Prototype

### 3.1. Laboratory Test of Laser Emission Pulse

**Figure 4:**
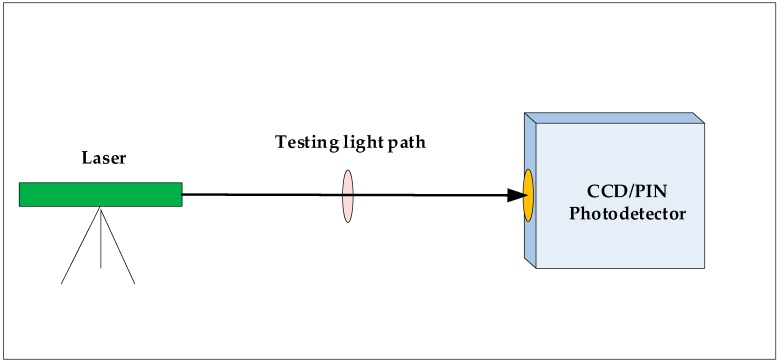
Laboratory test of laser emission pulse.

A PIN (positive intrinsic-negative) photodetector was used to measure the laser pulse waveform and the laser pulse duration in the laboratory (Figure 4). The laser pulse size and energy distribution characteristics were measured with the array CCD detector.

The results of the high-speed PIN photodetector test show that the full width at half maximum of the laser emission pulse is 1 ns. By using array CCD detector, the power/energy and the space of the laser emission pulse captured by the CCD are showed in Table 2, which reflects the quality of the laser emission spot is good. The laser beam on the *X*-axis and the *Y*-axis are Quasi-Gaussian pulses. Divergence angle of the laser beams is calculated by CCD camera technique measuring spot size, and the result is 5 mrad.

### 3.2. Laboratory Test of the Laser Echo Signal

**Figure 5:**
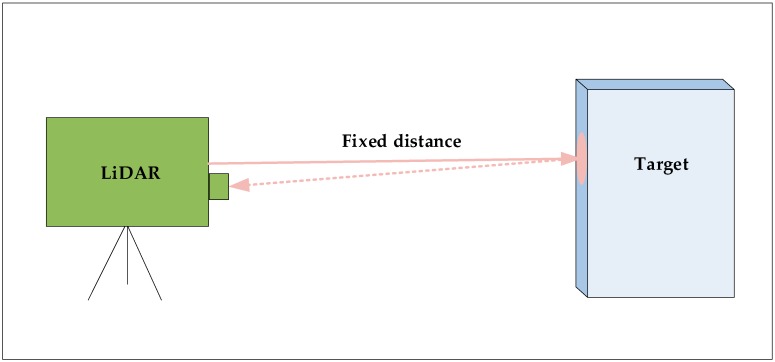
Laboratory test of laser echo signal.

A second laboratory test was performed to assess the distance measurement capabilities of the laser-sensor system. By pointing the laser at a fixed hard target with a distance of 944.1 m, the time delay between laser pulse emission and detection was measured (Figure 5). After more than 10 min of cumulative measurement, 450 pulses were randomly selected for analysis using MATLAB R2014a software manufactured by MathWorks Inc, Natick, MA, USA.

**Figure 6:**
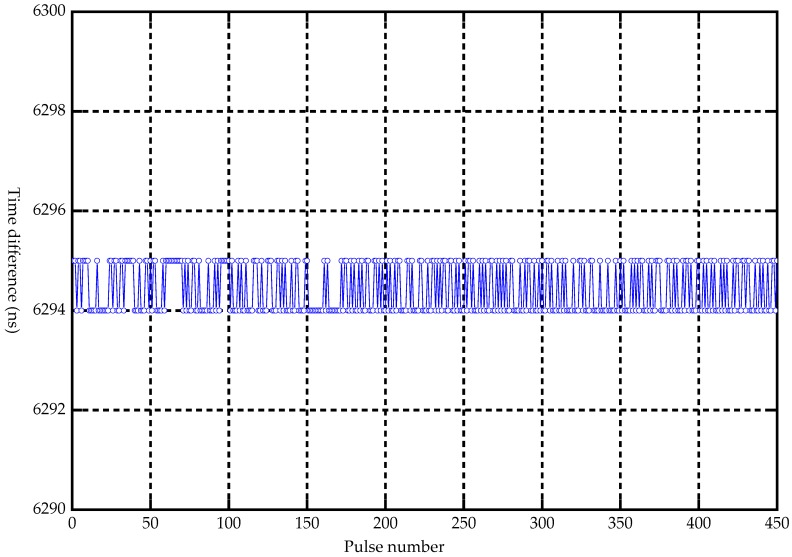
Time jitter result.

**Figure 7:**
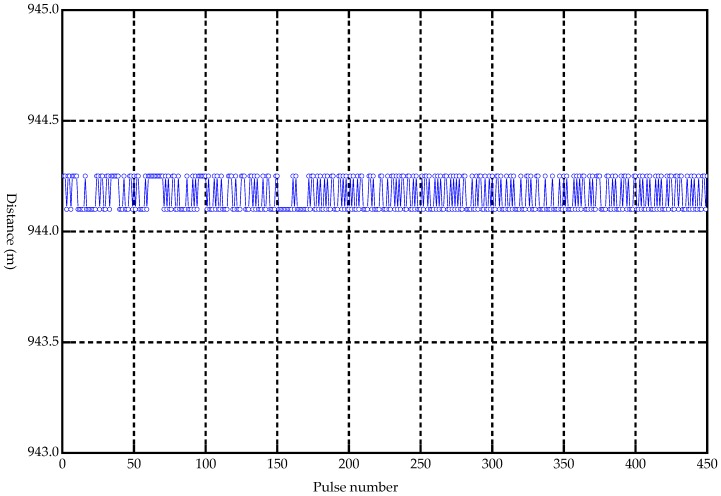
Corresponding distance measurement error.

The analyzed result based on MATLAB R2014a showed that the noise (jitter) of echo delay time was less than 1 ns (Figure 6), and the ranging accuracy was thus less than 0.15 m (Figure 7), which indicated that the large-footprint LiDAR equipment had good performance.

## 4. Experimentation Outdoors

### 4.1. Field Testing of the Instrument

As part of the calibration and validation activities for the TECM mission, LVDS was used in two operational tests, the first of which was conducted in Shijiazhuang, Hebei province in April 2017, and the second in the Northwestern Hunan province in December 2017. This paper only addresses results from the second test.

The aerial campaign for the second LVDS forest resources detection test began at the Lotus airport near Zhangjiajie city, which is in Hunan Province, in December 2017. Two flights were carried out. The first flight was between 10:52 and 15:35 on 24 December 2017. During the 283 min flight, 19 files containing a total of 28 GB of large-footprint LiDAR data were collected. The second flight occurred between 11:11 and 16:22 on 25 December 2017. During this 191 min flight, 43 files contained 33 GB of large-footprint LiDAR data were collected. The flight routes were chosen in order to cover landscape types and species assemblages of interest in Taoyuan, Huayuan, Baojing, Yongshun, Yuanling, and Guzhang counties, and Jishou City in Northwestern Hunan province, and were more than 980 km in length. The planes flew at elevations of 3000 m over the ground.

**Figure 8:**
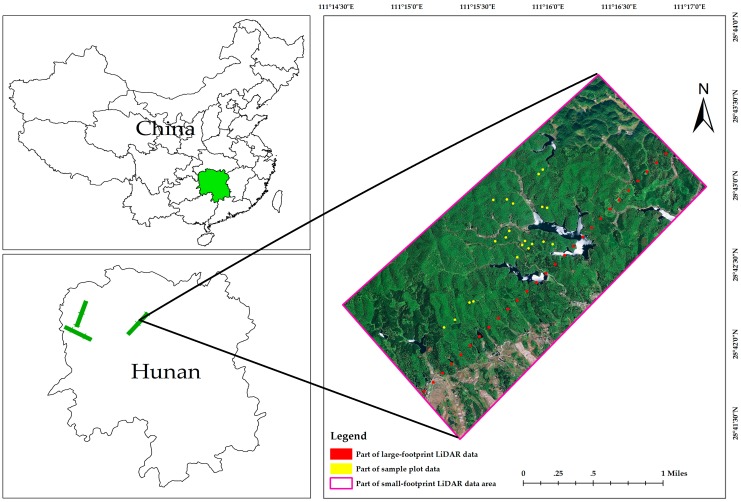
A segment of the flightline on which large-footprint LiDAR data (red points), small-footprint LiDAR (large rectangular area) and ground plots (yellow points) were collected.

In the field sample plot data collection, forest parameters including species type, canopy height, and diameter at breast height (DBH) were measured. A total of 306 circular sample plots with a diameter of 15 m (with an area of 177 m^2^) were measured in mixed forests between December 2017 and January 2018. The DBHs of all trees greater than 5.0 cm were measured in each plot. In order to improve the accuracy of the investigation [46], plot centers were geographically located using a Galaxy 6 RTK GNSS receiver manufactured by SOUTH Inc, Surveying and Mapping, Guangzhou, China. These units have a horizontal accuracy better than 0.25 m. For each of the sample plots, GNSS points were collected for more than 30 min using a one second logging rate. Forest canopy heights were measured using a VERTEX LASER VL5 manufactured by Haglof, Sweden [47]. These devices have a vertical accuracy of 0.1 m. All geographic data were collected using the WGS84 datum and the UTM zone 49N coordinate system (Figure 8).

#### 4.1.1. Estimation of Mean Forest Height Based on LVDS Data

The first step in the data analysis workflow for the forest height estimation tests was to pre-process the raw LVDS data. This included reducing the data volume through an approximately 1/100 subsample. First we removed acquisition sites that were not in forest or were not on flat ground, and then we subsampled the dataset to arrive at a total of 36 sites that met criteria in Taoyuan County; these were the points used in subsequent steps, including LVDS height estimate validation.

##### Large-Footprint LiDAR Data Processing

To calculate the mean forest height for each LVDS site, the waveforms were pre-processed, which included noise estimation, threshold calculation, identification of wave initiation, and bare ground identification. In order improve on processing speed, a 500 points subset around the maximum return value (200 points before and 300 points after) was chosen, as this range consistently contained the relevant information needed for canopy height estimation. Hereafter, all references to the waveform will be to this subset dataset.

(1) Noise estimation

Before the device detects the signal from the ground or the crown reflection, the background noise of the signal is received. After analyzing 200 waveforms, we developed the following heuristics with which to identify the location in the waveform at which the signal associated with reflectance from an object on the ground begins and ends. First, the mean and variance of the return energy associated with noise were estimated before the relevant part of the return signal by using the first 50 observations comprising the waveform. That is:

(1) $${\bar{N}}_{b}=\frac{\sum_{i=1}^{50}w_{i}}{50}$$

and:

(2) $$S_{N_{b}}=\sqrt{\frac{\sum_{i=1}^{50}{\left(w_{i}-{\bar{N}}_{b}\right)}^{2}}{49}}$$

where *w_i_* is the voltage value of the *i*th return value of the waveform. Next, the mean and variance of the noise after the relevant portion of the waveform signal were calculated by using the last 50 data points of the waveform signal:

(3) $${\bar{N}}_{e}=\frac{\sum_{i=451}^{500}w_{i}}{50}$$

and:

(4) $$S_{N_{e}}=\sqrt{\frac{\sum_{i=451}^{500}{\left(w_{i}-{\bar{N}}_{e}\right)}^{2}}{49}}$$

(2) Signal threshold calculation

The noise detection threshold *T* used for LiDAR signal analysis is commonly chosen by multiplying $\bar{N_{b}}$ and $\bar{N_{e}}$ by an appropriate number *n* of standard deviations [48]. The appropriate value of *n* varies in different studies (*n* = 2 [49], *n* = 3 [50], *n* = 3.5 [51], *n* = 4 [52], *n* = 4.5 [53]). In this study, *n* = 2 was used, i.e.,

(5) $$T_{b}=\bar{N_{b}}+2S_{N_{b}}$$

and:

(6) $$T_{e}=\bar{N_{e}}+2S_{N_{e}}$$

*T_b_* was used to extract the initial position of signal, while *T_e_* was used to extract the ground position.

(3) Estimating initial signal position

The initial position of the waveform signal (*S_b_*) represents the location of the highest tree in the study area. It defines the location of the signal’s starting position, and was defined as where the values of three consecutive frames (from the front to the back) are all larger than the begin-threshold value. That is, for each LiDAR return *w_j_* (*j* = 1…500):

*S_b_ = w_j_* if (*w_j-2_* < *T_b_* ∧ *w_j-1_* < *T_b_* ∧ *w_j_* < *T_b_*) is true (7)

(4) Estimating ground position

Due to a high proportion of canopy cover and low ground solar energy reflection in the study area, the return energy diminishes when the pulse is reflected by the ground. Using the same logic as above, it follows that the signal associated with the ground (*S_g_*) is where the values of three consecutive frames (from the back to the front) are all larger than the end-threshold value. That is, for each LiDAR return *w_j_* (*j* = 1...500):

*S_g_ = w_j_* if (*w_j-2_* > *T_e_* ∧ *w_j-1_* > *T_e_* ∧ *w_j_* > *T_e_*) is true. (8)

(5) Estimating mean forest height

**Figure 9:**
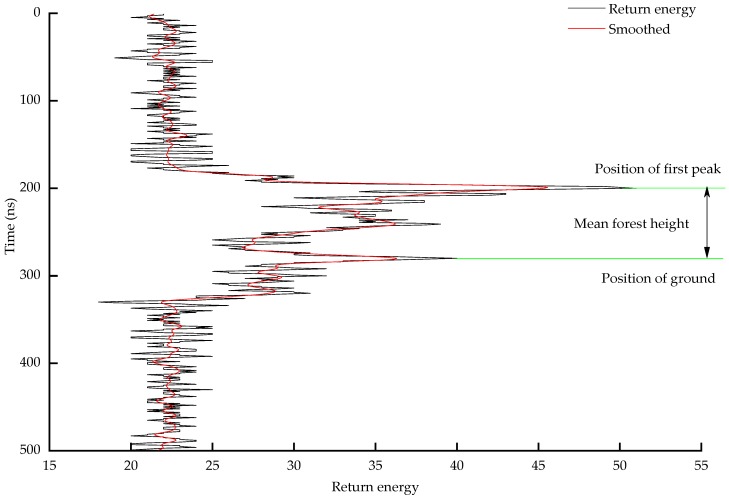
Estimation of mean forest height. The black line connects each of the 500 ns-level reflectance observations, and the red line is a filtered (fast Fourier transformation) representation of the same information.

Mean forest height associated with a laser pulse (*H_LVDS_*) is calculated by multiplying half of the distance light travels in one nanosecond (30 cm) by the difference between *S_g_* and *S_c_*. The initial signal position is the emission energy associated with reflection from the top of the canopy. As the canopy area increases, more and more energy is returned, and reaches the peak at the mean forest height location. Therefore, the first peak position (*S_c_*) after the initial signal position is defined as the mean forest height position. From the mean forest location and the ground location, mean forest height can be estimated as (Figure 9):

*H_LVDS_* = *R_v_*(*S_g_* − *S_c_*) (9)

*H_LVDS_* is the mean forest height and *S_g_* is the location of the recorded time of the ground position, *S_c_* is the recorded time of the mean forest location, and *R_v_* is a resolution of 15 cm (the signal can, thus, be written as the time interval of 1 ns, which gives a resolution of 15 cm.).

##### Height Modeling

**Figure 10:**
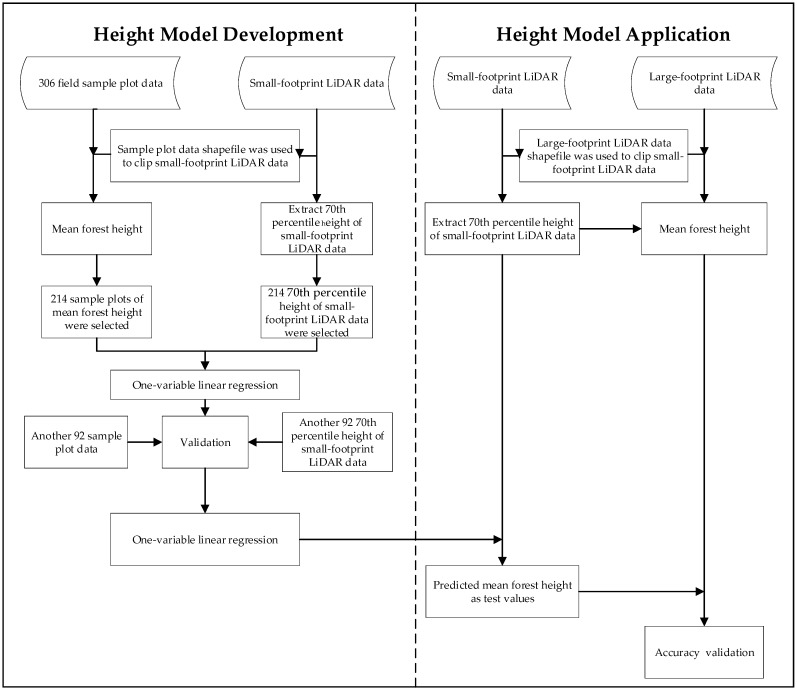
Overview of height modeling and validation process for the estimation of mean forest height.

The height modeling workflow is describe in overview in Figure 10. Although the center locations of the footprints for the large-footprint test site acquisitions were georeferenced, it was impossible to collocate our ground plots with the large footprint LiDAR sites (e.g., Figure 8) in order to calibrate height models or validate estimates. Therefore, we used height estimates modeled from small-footprint LiDAR data collected from another study as inputs to the LVDS validation process. Small-footprint LiDAR data offers the unique opportunity to bridge the gap between large-footprint LiDAR data and sample plot data. The existing small-footprint LiDAR data with which we had to work consisted of a 7 km × 90 km study site that covered the LVDS acquisition area. These data were collected on 20 December 2017 with a point density of 5.3 returns/m^2^. Georeferenced point clouds containing information on height above ground level were created using standard processing methods.

GIS files of the ground sample plot footprints (where tree heights were measured) were used to create subsets of the small-footprint LiDAR data contained within them. For each of several candidate small footprint LiDAR within-plot point cloud height value percentiles, simple linear regression models of the relationship between sample plot-based mean forest height (dependent variable) and small footprint LiDAR height value percentiles (independent variable) were constructed using 306 ground plots. A total of 214 of the 306 samples (96 samples whose dominant species were coniferous and 118 samples whose dominant species were deciduous) were used as the training samples and another 92 samples (41 coniferous and 51 deciduous) were treated as test samples for accuracy evaluation. After assessing model fit statistics for the candidate point cloud height percentiles, the 70th percentile point cloud height value was selected as a surrogate for measured mean forest height on a plot, and the associated regression models estimating forest height from the small footprint LiDAR point cloud data were used in subsequent analysis.

Mean forest height was estimated using the linear regression model from the previous step within the boundary for each of the 36 LVDS sites in our test. To assess accuracy of the height model, Mean Absolute Deviation (MAD) and mean absolute percentage error (MAPE) were calculated as follows:

(10) $$\mathrm{MAD}=\frac{1}{n}\overset{n}{\underset{i=1}{\sum }}\left|x_{i}-\bar{x}\right|$$

and:

(11) $$\mathrm{MAPE}=\frac{1}{n}\overset{n}{\underset{i=1}{\sum }}\left|\frac{x_{i-}\bar{x}}{x_{i}}\right|\cdot 100$$

where $x_{i}$ is the deviation (ground plot canopy height—70th percentile height) associated with plot *i* and $\bar{x}$ is the average of $x_{i}$ across all 36 observations.

##### Comparison of LVDS and Height Modeled from Small Footprint LiDAR Regression Equations

The chosen linear model of the regression of mean forest height (H_SFL_) on the 70th percentile height value of the small-footprint LiDAR data ($X_{70^{\mathrm{th}}})$ was of the form

(12) $$H_{SFL}=0.8822\cdot X_{70^{\mathrm{th}}}+2.3147$$

**Figure 11:**
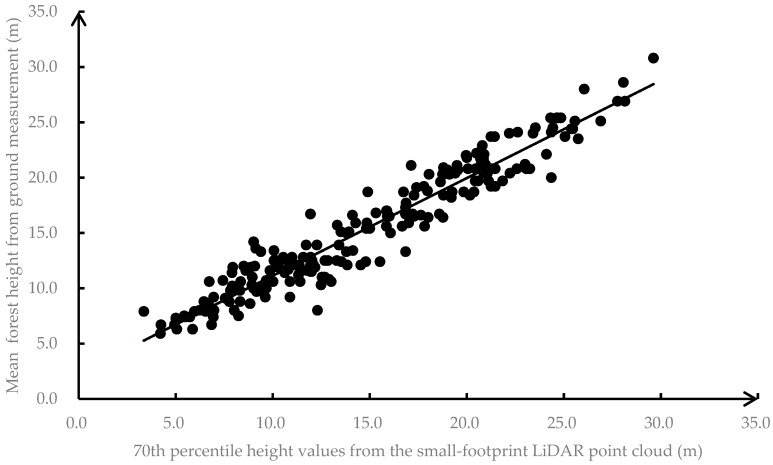
Relationship between 70th percentile small-footprint LiDAR height values and heights measured on sample plots (*n* = 214). The dark line is the linear regression line from Equation (12).

The model’s R^2^ was 0.92 and the root mean square error (RMSE) was 1.58 m (Figure 11).

Data from 92 sample plots not used to develop the height model were used to evaluate the estimation accuracy of Equation (12). Using deviations calculated from the difference between the LiDAR-estimated and actual (ground) heights at these 92 locations, the MAD was found to be 1.32 m and the MAPE was found to be 9.4%. It is difficult to say, however, if the deviations occurred due to inaccuracies in the ground height measurements or the small-footprint LiDAR data aggregation approach. The relationship in Figure 11 does appear to be linear, however, with a nearly 1:1 relationship and no evidence of heteroscedasticity, suggesting that the model performance is acceptable.

**Figure 12:**
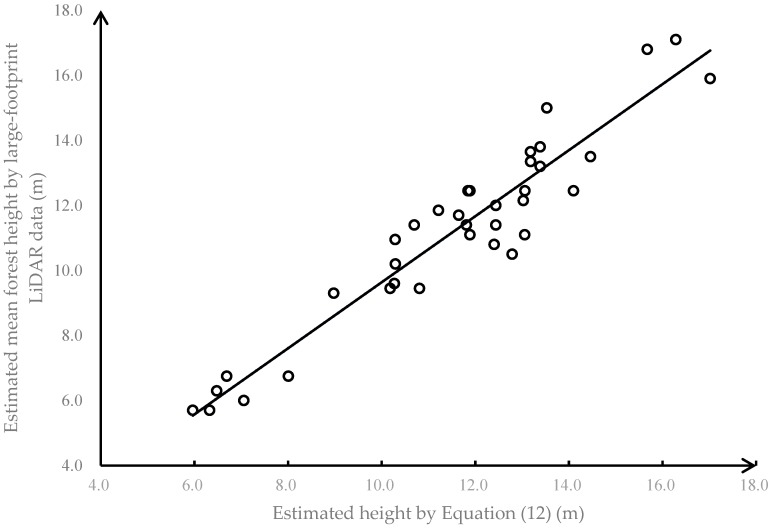
Mean forest height comparison between estimated by Equation (12) and estimated by large-footprint LiDAR data.

In order to validate height estimates derived from the LVDS instrument (Equation (9), Figure 9), Equation (12) was applied to the 70th percentile of the small footprint LiDAR height values associated with the polygonal LVDS footprints (a 15 m diameter circle) for the 36 validation plots. In other words, the 70th percentile of the small footprint LiDAR height values within the LVDS validation plot footprint was calculated and compared to the LVDS-derived height estimate. Results are shown in Figure 12. The MAD was 0.78 m, and the MAPE was 6.8%. The relationship does follow the 1:1 line, and there does not appear to be any systematic bias or heteroscedasticity. These results strongly suggest that the LVDS system performs as expected when measuring forest canopy height, and can be a valuable tool for monitoring both the status of and trends in forest cover in China and elsewhere.

## 5. Discussion

### 5.1. The Advantage of the Equipment

As with other studies conducted with the same type of equipment [30], an advantage of this approach is the integration of a large laser spot emission and a large field of view. A laser beam is used to couple the laser pulse to a beam splitter through an optical fiber. The part of the beam with less energy is coupled to the relay optical device through the optical fiber. The remainder of the beam, which has more energy, is sent to the ground target after it is expanded. The placement of the refractive mirror in front of the telescope shortens the system length, collimating and focusing the reflected signal and maintaining its strength, improving the detection efficiency of the system. The light path through the detector uses a custom prism, allowing for the appropriate attenuation of the signal to correspond with the limits of the detector. In our system design, the combination of this prism and laser output attenuator thus adjusts the trigger energy.

In addition, the device adopts a high-precision time difference measurement mode governed by the transmitting and receiving optical paths. The laser emitting pulse interacts with the beam splitter through the optical fiber. The beam splitter lens diverts a small energy emitting trigger laser beam to a relay optical device through an optical fiber. The relay optical device sends the input emitting laser trigger pulse along the detector trigger light path. By using a custom prism, the transmitting pulse and the echo signal received by the telescope are coupled to detect the transmitted light pulses and echo signals directly, which avoids errors in the measurement of time difference caused by some factors, like laser dithering and system timing error, thus improving the accuracy of system time and distance measurement.

### 5.2. The Positional Accuracy of the Sample Plots

Some studies use handheld GPS units to locate sample plot centers [54]. It is generally known that with many commercially-available GPS units, the horizontal error is about 5 m where the signal is good [55,56]. In dense forest, the error may reach 30 m or more [57]. Therefore, if using handheld GPS to locate the sample plots with the aim of collocating them with the footprints of aerial laser pulses, large errors can occur. Real-time positioning that relies on GNSS units can be unreliable as well; when used outside the signal range, GNSS units’ positioning accuracy is not much different from that of a normal handheld GPS.

In our study, the total station and GNSS are used together to determine the position of sample plot center and all trees in the sample plot. First, GNSS1 was set up in a place with good mobile phone signal. After arriving at the sample plot site, the total station was set up at the center of the selected surveyed sample plot. The origin of coordinates of the total station was set as (0,0,0). The direction was selected and the virtual coordinate system was established. At the same time, GNSS2 and GNSS3 were set up in the relatively empty position of the sample plot, and the GNSS2/3 could clearly be seen by the total station. All the three GNSS base station units were selected static mode. All GNSS base stations worked at least 30 min at a time, and no more than 30 km apart. In addition, GNSS1 can obtain highly accurate location coordinates by selecting CORS (continuously operating reference stations) service. Taking the high accuracy coordinate as the control point, the differential data was processed. GNSS2 and GNSS3 within the sample plot have both the virtual coordinates collected by the total station and the high accuracy coordinates after the differential data processing. Through the coordinate transformation relationship, the accurate coordinates of the sample plot center and all trees can be obtained.

In other words, through setting up three GNSS base station units (one of which is located using the CORS service), the sample plot positional accuracy was greatly enhanced.

### 5.3. Accuracy of Large Laser Spot Data Validation

Due to the influence of the sensor itself, atmospheric scattering, and other factors, a certain amount of noise often exists in the received echo waveform. The accuracy of noise estimation and waveform data processing algorithms affects the estimation of mean forest height. Laboratory tests showed that the measurement jitter of echo delay time was less than 1 ns, and the range precision was better than 0.15 m for the measurement distance of a fixed target (Section 3.2). Based on these laboratory tests, customized noise detection thresholds were determined. Future research will assess the universality of these thresholds under different operating conditions.

Previous studies on extracting mean forest height included areas with sloping topography [39,58,59]. However, based on the need for a timely performance verification of the LVDS system, we devised a new method of calculating mean forest height over level terrain. It is worth noting that the method proposed in this paper is thus mainly suitable for level areas. Terrain slope will have an impact on the pulse broadening [52], thus directly affecting height estimation. Therefore, future research will account for this and include assessing the performance of the newly designed system in areas with undulating topography.

## 6. Conclusions

Our study of the LVDS system provides a test platform for the large-footprint LiDAR system to be used on the TECM satellite-borne LiDAR mission to be launched in 2020. We demonstrated through both laboratory testing of the individual components and field testing of the integrated system that the height measurements provided by the system are acceptable. The deviance between the instrument’s height measurements and those obtained in the field are small, and the impacts of this deviance on tree biomass estimation is small. We, therefore, conclude that data from the sensor system has much potential to improve forest monitoring and other land cover assessment projects in China and elsewhere.

## Figures and Tables

**Table 1:** iXU-R180 technical index.

Index Name	Design Value
Resolution	10,328 × 7760 (80 MP)
Dynamic range	>72 db
Pixel size	5.2 µm
CCD size effective	53.7 mm × 40.4 mm
Aspect ratio	4:3
Light sensitivity (ISO)	35–800
Shutter speed	1/1600 s
Camera lens focus	50 mm f/4.0
FOV	56.5° × 44°
Interfaces	USB 3.0
Minimum photo interval	1.8 s
Data storage	1 TB SSD storage (optional iX Controller) CompactFlash card Type I/II including UDMA 6 and 7

**Table 2:** Power and space of laser emission pulse.

Name	Value	Unit
Power/energy		
Total energy	445,221,505.16	cnts
Peak value	8777.03	cnts
Minimum value	−205.77	cnts
Space		
The coordinate of centroid on the *X*-axis	1.484627 × 10^4^	μm
The coordinate of centroid on the *Y*-axis	1.230543 × 10^4^	μm
D4 σ X	2.744 × 10^4^	μm
D4 σ Y	2.019 × 10^4^	μm
